# Supplementary material for: Chromothripsis during telomere crisis is independent of NHEJ, and consistent with a replicative origin
Source: Genome Res. 2019 May;29(5):737–49. doi: 10.1101/gr.240705.118 (PMC6499312; doi:10.1101/gr.240705.118)
Supplement: Supplemental Material [file supp_gr.240705.118_Supplemental_file_1.zip › contigs/annotated_contigs/DB53/contig.2.DB53_length_733_mean_cov_8.99454297408.docx]

**DB53_length_733_mean_cov_8.99454297408**

```
AAGAGATTAATGAAGATTATAGGGTGATTAAGGCTTTCATCCATGTTCTCAGTTGCCACTATCACTGATGGCAACAAAGTTACAATGTG
 >chr10:57688830-57689180 - E=9e-199
AATTTAAACATAGCTTTTTTAACTTGAATCTGCTTCCCTTCTTTTCTTGGAATACTAAAACACTAACATTCCCAAAACATCCATTTATA

TTAATAGAGTTAAGTAAAGGGTGTATAAACAGGAACACAATAATTCTTCTAAGTTATACCTTATCTACTGTATTATGTAAAGCCAAGTA

AACTTATTTCAAAAAAGAAGGAATTGGATTTTGCAAAAGATTAAAATAATTTTGTACCATCAAACTCTATCAAACTTCTGCT|T|GATT
                                                                                   >chr
TTGTCATGCACGTCCATGTGAAGAGACCACCAACAGGCTTTGTGTGAGCAATAAAGCTTTTTAATTACCTGGGAGCAGGTGGGCTGAGT
10:57736161-57736523 - E=6e-191
CCAAACAGAGAGTCAGCAAAGGGAGATAGGGTGGGGCAGTTTTATAGGATTTGGGTGGGTAGTGGAAAATTACAGCCAAAAGGGGTTTT

TCTCTTGCAGGCAGGGGTGGGGGTCACCAGGTGTTCAGTAGAGGGAACTTCTGAGCCAGGAGAAGGAATTTCACAAGGTAATGTCATCA

GTTAAGGCAGGAACCGGCCATTTTCACTTCTTTTGTGATTCTCCAATTTCTTCAGGCCAATTGGAGGTACACATGCAGGGTTGGTCTCA

G|GGGTCGAACCGTTCGCACTAAA
```
